# Hypergravity Stimulation Enhances PC12 Neuron-Like Cell Differentiation

**DOI:** 10.1155/2015/748121

**Published:** 2015-02-16

**Authors:** Giada Graziana Genchi, Francesca Cialdai, Monica Monici, Barbara Mazzolai, Virgilio Mattoli, Gianni Ciofani

**Affiliations:** ^1^Istituto Italiano di Tecnologia, Center for Micro-BioRobotics @SSSA, Viale Rinaldo Piaggio 34, 56025 Pontedera (Pisa), Italy; ^2^ASAcampus Joint Laboratory, ASA Research Division, Department of Experimental and Clinical Biomedical Sciences, University of Florence, Viale Pieraccini 6, 50139 Florence, Italy

## Abstract

Altered gravity is a strong physical cue able to elicit different cellular responses, representing a largely uninvestigated opportunity for tissue engineering/regenerative medicine applications. Our recent studies have shown that both proliferation and differentiation of C2C12 skeletal muscle cells can be enhanced by hypergravity treatment; given these results, PC12 neuron-like cells were chosen to test the hypothesis that hypergravity stimulation might also affect the behavior of neuronal cells, in particular promoting an enhanced differentiated phenotype. PC12 cells were thus cultured under differentiating conditions for either 12 h or 72 h before being stimulated with different values of hypergravity (50 g and 150 g). Effects of hypergravity were evaluated at transcriptional level 1 h and 48 h after the stimulation, and at protein level 48 h from hypergravity exposure, to assess its influence on neurite development over increasing differentiation times. PC12 differentiation resulted strongly affected by the hypergravity treatments; in particular, neurite length was significantly enhanced after exposure to high acceleration values. The achieved results suggest that hypergravity might induce a faster and higher neuronal differentiation and encourage further investigations on the potential of hypergravity in the preparation of cellular constructs for regenerative medicine and tissue engineering purposes.

## 1. Introduction

Altered gravity represents a powerful physical cue able to exert deeply modeling effects on both anatomy and function of living organisms [1–10]. It is indeed well known that exposure to microgravity implies detrimental effects on skeletal muscle mass [11], composition [12], and contractility [3], and on bone density, with long-term effects even after return to normal gravity [13]. Microgravity instead has contrasting effects on the nervous system, in some cases not perturbing cell differentiation and assembling [14–16], in other cases strongly altering cell morphology and functions [17–19] or even improving stem cell differentiation into neurons [20–22].

For its demonstrated effects from the cellular level up to the whole organism level, microgravity has been proposed in regenerative medicine for a wide range of applications, including self-assembling of healthy and diseased tissues, and drug testing. [23–26]. For instance, simulated microgravity was applied to bone marrow stromal cells for transplantation in contusion model mice, significantly improving motor function [27]. PC12 adrenal medullary cells were tested under microgravity conditions as well, and neuroendocrine organoids were thus successfully achieved [28].

Many other examples focused on simulated microgravity for both basic science investigations and regenerative medicine purposes can be found in the literature, whereas few reports deal with hypergravity effects on biological systems. Among these, it is worth to mention a study on the vestibular system of* Oryzias latipes*, demonstrating an early upregulation of the* c-fos* gene transcription as soon as 30 min after a 3 g exposure [5]. Another study showed that a 2.9 g hypergravity treatment proved to inhibit bone resorption in ovariectomized rats [7]. Other experiments on cellular systems were performed on human umbilical vein endothelial cells (3.5 g for 96 h [29]) and on SH-SY5Y neuroblastoma cells (2 g over 6 min [30]), respectively, showing an altered cytoskeletal distribution and an enhanced lamellar protrusive activity. Concerning hypergravity effects on skeletal muscle, C2C12 myoblast proliferation and differentiation were shown to be significantly increased by 5, 10, and 20 g treatments [31]. In the field of tissue engineering, it has been shown that hypergravity helps to promote a higher expression of cardiomyocyte proteins in mesenchymal stem cells and that the latter can be successfully grafted in a mouse model of cardiac infarction supporting an improved functional recovery [32].

As evident in most of the reported examples, low hypergravity values (1–10 g) have been usually studied for their implications on human health, being those typically acting on astronauts in the early stages of the journey from the Earth to space. A wider range of hypergravity values has instead poorly been investigated, leaving largely undisclosed hypergravity potentialities for both basic and translational science. The exploration of strong hypergravity values indeed opens opportunities to a better understanding of cellular responses to extreme conditions, and to the preparation of constructs with desired phenotype for cell transplantation and drug screening procedures.

In the light of the previously mentioned studies, and aiming at developing new strategies in the field of neuronal tissue engineering, it was hypothesized that hypergravity might also affect PC12 neuron-like cell behavior, in particular during differentiation. For these reasons, high acceleration values (50 g and 150 g) were chosen for hypergravity stimulation and applied for 1 h at different time intervals since the differentiation induction. Several qualitative and quantitative analyses (fluorescent staining, metabolism assay, gene expression analysis) were performed, highlighting as the hypergravity stimulation caused a slight increment of metabolic activity, but also a more marked enhancement of neuronal differentiation. These results demonstrate as hypergravity efficiently works as a straightforward physical stimulation for biomedical applications, and it can have important implications in the field of peripheral nerve regeneration.

## 2. Materials and Methods

### 2.1. Cell Culture

PC12 cells (derived from a rat pheochromocytoma) represent a model for neuronal differentiation, as they can reversibly respond to the administration of the nerve growth factor (NGF) and express a sympathetic neuronal phenotype [33]. PC12 cells were seeded at a density of 20,000 cells/cm^2^ on tissue culture polystyrene (PS) disks, previously coated with a 100 *μ*g/mL collagen I (Sigma) solution to promote cell adhesion. PC12 were cultured in Dulbecco's modified Eagle medium (DMEM), supplemented with 10% horse serum (HS), 5% fetal bovine serum (FBS), 100 IU/mL penicillin, 100 *μ*g/mL streptomycin, and 2 mM L-glutamine. Differentiation was induced by administration of a low-serum (1% FBS) medium, supplemented with 50 ng/mL NGF (Sigma). PC12 cells were maintained at 37°C in a 5% CO_2_, saturated humidity atmosphere. Adhesion and proliferation were allowed for 48 h before performing experiments.

**Figure 1:**
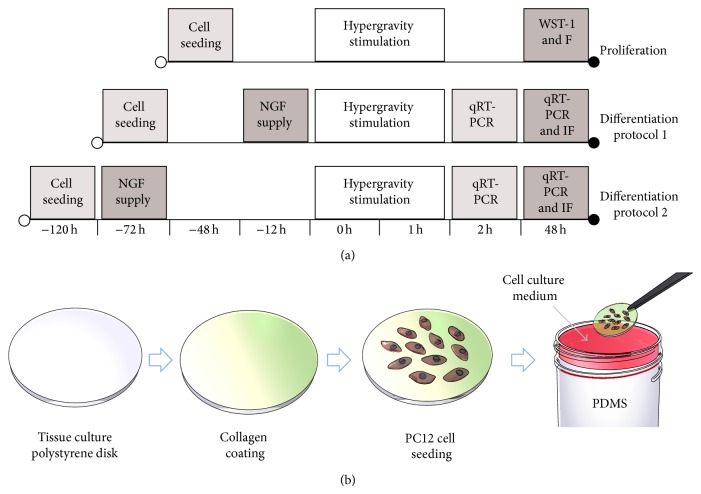
Experimental timeline. “F” stands for “fluorescent staining” and “IF” stands for “immunofluorescent staining” (a). Schematization of the setup used to expose PC12 cells to hypergravity (b).

Differentiation was induced for two different time intervals (12 h and 72 h) before applying stimulation, in order to evaluate the effect of hypergravity on phenotype at different development stages. A predifferentiation period of 12 h was chosen to assess the effect of hypergravity on the early events of neuritogenesis, whereas a predifferentiation period of 72 h was chosen to study any perturbations on later stages of neuritogenesis [34]. Experimental protocols are schematized in Figure 1(a).

For hypergravity stimulation, PS disks seeded with PC12 cells were placed over cured polydimethylsiloxane (10 : 1 base to cross-linking agent ratio, curing temperature: 60°C), filling 90% of the volume of 50 mL centrifuge tubes. The remaining 10% of the volume of the tubes was filled with cell culture medium, in order to exclude shear stress effects during stimulation (Figure 1(b)). Hypergravity (50 g and 150 g) was applied with a bench centrifuge (312R Hettich Zentrifugen, equipped with a 1617 rotor) thermostated at 25°C for 1 h. Control cultures were kept at 1 g and 25°C for 1 h. At the end of hypergravity stimulation, cultures were transferred to 24-well plates and kept under either proliferating or differentiating conditions before being analyzed.

Analyses were mostly performed 48 h after hypergravity treatment, as a trade-off between the timeframe usually needed to observe effects of treatments on PC12 cell phenotype under differentiating conditions [35] and the potential occurrence of recovery effects after hypergravity stimulation [36]. However, analyses on gene transcription were performed also 1 h after hypergravity treatment as alterations at gene transcriptional levels can occur even at extremely early time points after treatments.

### 2.2. PC12 Cell Proliferation

Analyses were performed 48 h after hypergravity stimulation. Cell proliferation was assessed with the WST-1 cell metabolism assay (based on the 2-(4-iodophenyl)-3-(4-nitophenyl)-5-(2,4-disulfophenyl)-2H-tetrazolium monosodium salt, provided in a pre-mix electro-coupling solution, BioVision). Cell cultures were treated with 400 *μ*L of culture medium added with 40 *μ*L of pre-mix solution for 2 h and, finally, absorbance was read at 450 nm with a microplate reader (Victor X3, Perkin Elmer).

### 2.3. Fluorescence Staining of Proliferating and Differentiating Cultures

Fluorescence staining was performed on cultures 48 h after hypergravity stimulation. Samples were first rinsed with 1X PBS (PBS) and fixed with a 4% paraformaldehyde (Sigma) solution in PBS for 20 min at 4°C. After three rinses with PBS (5 min each), samples were incubated with 1 mg/mL sodium borohydride (Sigma) in PBS for 10 min to reduce or suppress aspecific fluorescence. Cellular membranes were then permeabilized with 0.1% Triton X-100 (Sigma-Aldrich) in PBS for 15 min. Aspecific binding sites were saturated with 10% goat serum (GS, Gibco) in PBS for 1 h. Cell morphology of proliferating cultures was investigated by staining cytoskeletal actin and nuclei with a 60 ng/*μ*L TRITC-phalloidin (Millipore) solution and a 1 *μ*M DAPI solution in 10% GS, respectively. After three rinses in PBS (5 min each), samples were imaged with a confocal laser scanning microscope (C2 s, Nikon). For imaging of neurites in differentiating cultures, a rabbit polyclonal antitubulin IgG (Sigma, diluted 1 : 75 in 10% GS), or a rabbit monoclonal antineurofilament IgG (Millipore, diluted 1 : 200 in 10% GS) were used as primary antibodies after saturation on different sets of samples (stimulated with the same hypergravity conditions). Cultures were incubated with primary antibodies at 37°C for 45 min and then washed four times with 10% GS (5 min each rinse). A fluorescent goat anti-rabbit IgG (Invitrogen, diluted 1 : 250 in 10% GS) was then used as a secondary antibody, with an incubation at room temperature for 30 min, while nuclei were counterstained with a 1 *μ*M DAPI solution in 10% GS. Unbound and weakly bound antibodies were removed with 0.45 M NaCl PBS (1 min rinse). Samples were finally washed twice with PBS (5 min each rinse) and images were acquired with the confocal microscope.

Neurite lengths were measured with ImageJ software. For statistical purposes, at least 30 neurites* per* culture were considered. Neurofilament-positive neurites and nuclei were identified with ImageJ software as well, considering more than 500 cells* per* sample type.

### 2.4. Gene Transcription Analysis: qRT-PCR

Investigations on gene transcription were performed 1 h and 48 h after hypergravity treatment. Total RNA was isolated from cell cultures by using High Pure RNA Isolation kit (Roche) according to the manufacturer's protocol. RNA concentration was measured at 260 nm with a spectrophotometer (NanoDrop 2000, Thermo Scientific). Retrotranscription of 300 ng of RNA into cDNA was performed by using the iScriptTM Reverse Transcription Supermix (Bio-Rad). The thermal protocol included an incubation at 25°C for 5 min, followed by an incubation at 42°C for 45 min and a final incubation at 85°C for 5 min to stop the reaction. Then, cDNA was diluted 10 times with ultrapure water to undergo amplification.

Neurofilament-66 (*Ina*) and *β*3-tubulin (*Tubb3*) gene transcription was analyzed through quantitative RT-PCR with a CFX Connect Real-Time PCR Detection System (Bio-Rad). Glyceraldehyde 3-phosphate dehydrogenase (*Gapdh*) was considered as reference gene. For PCR procedures, cDNA (5 *μ*L) was mixed to specific forward and reverse primers (at a final concentration of 8 *μ*M) and to SsoAdvanced SYBR Green Supermix (Bio-Rad). The following thermal protocol was applied: one period of 30 s at 98°C for polymerase activation, followed by 40 cycles at 98°C for 3 s and 60°C for 15 s. After the last reaction cycle, melting curves were obtained through a temperature ramp from 65°C to 95°C, with 0.5°C/s increments, to exclude unspecific products. Each PCR run included “no template” sample and all tests were performed in triplicate. The cycle threshold (Ct) value relative of control sample was considered for the calculation of ΔΔCt (difference between ΔCt values calculated from the difference between Ct of target and of reference gene) for the samples. Primer sequences (forward and reverse) of the investigated genes are reported in Table 1.

### 2.5. Statistical Analysis

Each experiment was repeated three times and a number of three samples were considered for each experimental session. Data were presented as mean ± standard deviation. Analysis of variance (ANOVA) was performed, followed by Bonferroni's* post hoc* test. Neurite length distributions were shown on box-plots and expressed as median ±95% confidence interval; Kruskal-Wallis analysis followed by Dunn's* post hoc* test was performed to test for significance. A *P* value < 0.05 was considered significant.

## 3. Results

**Figure 2:**
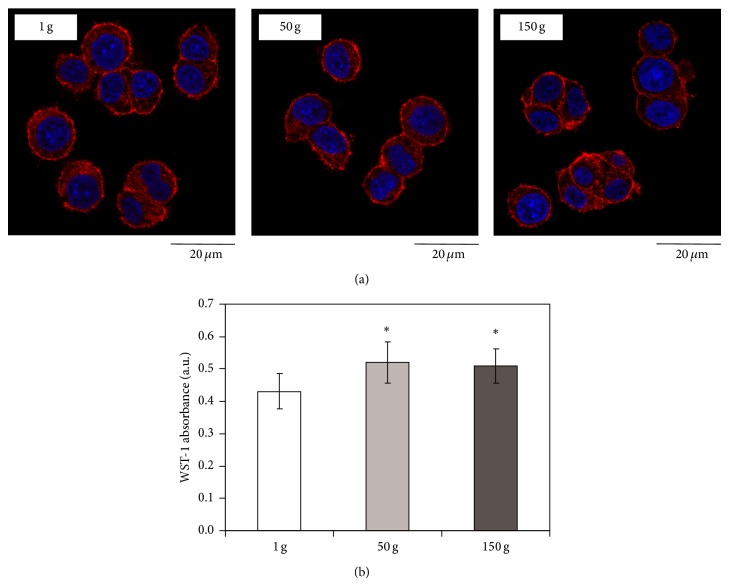
Confocal images of cytoskeletal actin (in red) and nuclei (in blue) in proliferating PC12 cells 48 h after hypergravity treatment (a); quantification of cell viability through WST-1 assay after hypergravity treatment (b). ^*^ *P* < 0.05.

Under proliferating conditions, cell morphology after hypergravity stimulation was found qualitatively comparable to that observed in control cultures; as shown in Figure 2(a), cells retained their typical round morphology with very small protrusions and were homogeneously attached to the substrate after both 50 g and 150 g treatment.

Instead, cell metabolism was found slightly but significantly higher (of about 20%) after hypergravity application with respect to the control cultures (Figure 2(b)).

**Figure 3:**
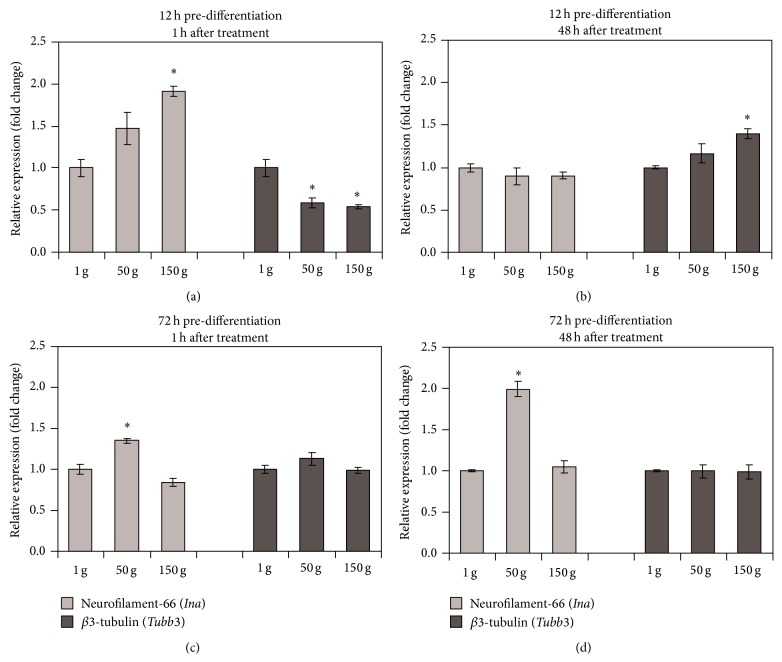
qRT-PCR results for neurofilament-66 (*Ina*) and *β*3-tubulin (*Tubb3*) gene transcription 1 h (a, c) and 48 h (b, d) after hypergravity treatment on PC12 cells predifferentiated for 12 h (a, b) and 72 h (c, d). ^*^ *P* < 0.01.

Concerning transcriptional levels of differentiation marker genes in cells differentiated for 12 h prior to hypergravity stimulation, a significant upregulation of* Ina* (1.9 fold at 150 g) and downregulation of* Tubb3* gene (1.7-fold at 50 g and 1.8-fold at 150 g) transcription were found immediately (1 h) after the treatment (Figure 3(a)). The transcription of* Tubb3* appeared upregulated after 48 h for the higher acceleration tested, being 1.4-fold in comparison to the control (Figure 3(b)).

In cells differentiated for 72 h prior to hypergravity stimulation, an upregulation of* Ina* (1.3 fold at 50 g) was found immediately after the treatment (Figure 3(c)). This upregulation became even higher (2-fold at 50 g) after 48 h (Figure 3(d)). All of the transcriptional levels of* Tubb3,* instead, remained constant in cells predifferentiated for 72 h.

**Figure 4:**
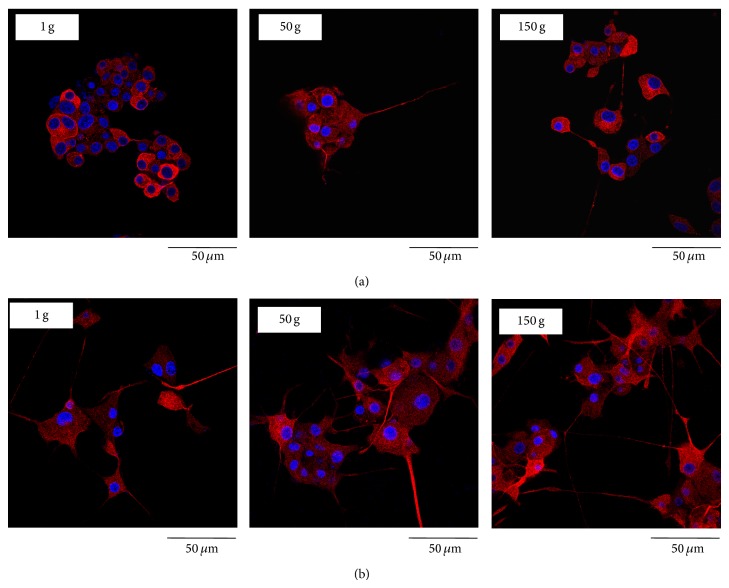
Confocal images of *β*3-tubulin (in red) and nuclei (in blue) in PC12 cell cultures predifferentiated for 12 h (a) and 72 h (b) 48 h after hypergravity treatment.

Immunofluorescent staining of *β*3-tubulin was performed on cultures predifferentiated for either 12 h (Figure 4(a)) or 72 h (Figure 4(b)) with NGF, 48 h after hypergravity stimulation. The most significant effects of hypergravity were represented by evidently longer *β*3-tubulin positive neurites in cells exposed to 150 g stimulation with respect to those at lower acceleration values. As shown in Figure 4(b), this evidence was more pronounced in the cultures predifferentiated for 72 h, where PC12 cells seemed to have a higher length and number of neurites after 150 g stimulation.

**Figure 5:**
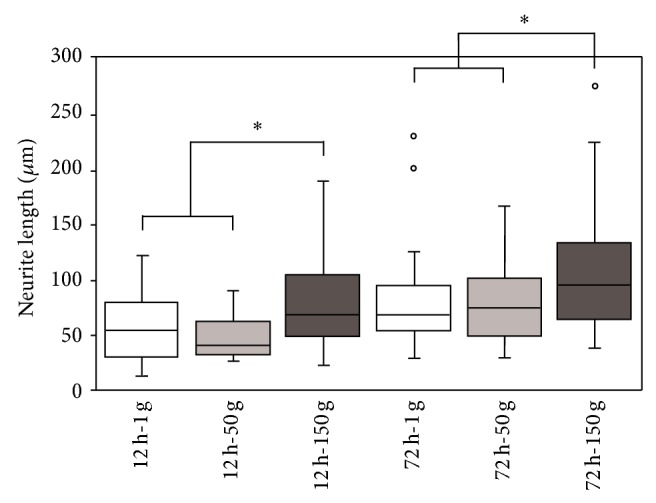
Box plot of the neurite lengths measured 48 h after hypergravity treatment. PC12 cells were differentiated for 12 h and 72 h prior to hypergravity treatment. ^*^ *P* < 0.05.

After *β*3-tubulin staining, the neurite lengths were measured by using low magnification fluorescence images (representative fields are provided as Supplemental Data, see Figure S1 in the Supplementary Material available online at http://dx.doi.org/10.1155/2015/748121), and their distribution was analyzed. As shown in Figure 5 with the box plot of the neurite lengths, the 150 g hypergravity treatment was effective at determining the emission of significantly longer neurites in PC12 cells predifferentiated for 12 h with respect to the lower acceleration values. Specifically, median neurite lengths were about 55 *μ*m (for 1 g); 40 *μ*m (for 50 g); and 70 *μ*m (for 150 g; *P* < 0.05) in cells predifferentiated for 12 h.

Even in cells predifferentiated for 72 h, the 150 g treatment promoted the sprouting of longer neurites with respect to the lower acceleration values. Median neurite lengths were about 70 *μ*m (for 1 g); 80 *μ*m (for 50 g), and 100 *μ*m (for 150 g; *P* < 0.05). Neurite length distributions are also reported as frequency histograms in Supplemental Data (Figure S2).

**Figure 6:**
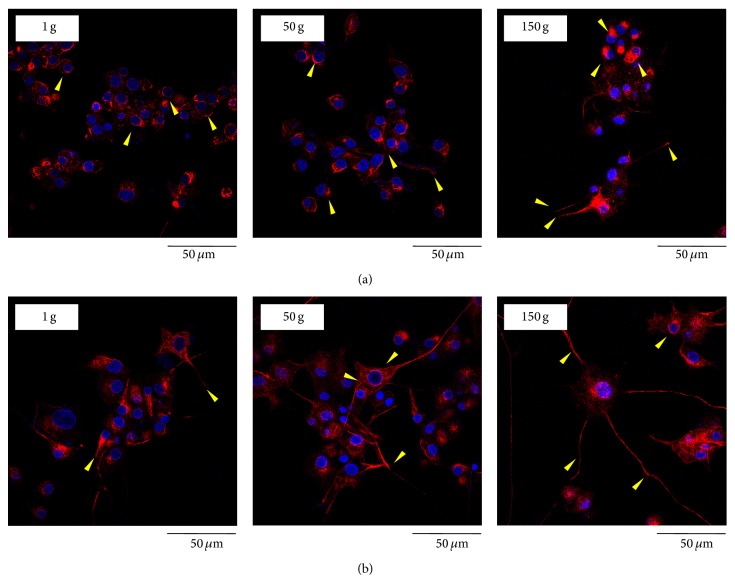
Confocal images of neurofilament-66 (in red) and nuclei (in blue) in PC12 cell cultures predifferentiated for 12 h (a) and 72 h (b) after 48 h from hypergravity treatment. Arrows evidence neurofilament-66 organization in a ring-like structure in control cultures, whereas the marker is localized in neurites and in growth cones in hypergravity-stimulated cells.

In control cells predifferentiated for 12 h, neurofilament-66 protein (as evidenced by immunofluorescent staining) surrounded nuclei as a neat ring-like structure, and a few neurites resulted positive to staining. In the cultures that underwent 50 g and 150 g stimulation (Figure 6(a)), neurofilament-66 showed a neat polarization on one side of the cell nucleus and a peculiar arrangement in the neurites, where the staining appeared in the distal portions and in the growth cones. A similar trend was found in cultures that underwent hypergravity stimulation after a 72 h predifferentiation period. However, in this case, neurofilament-66 polarization was present also in control cells (Figure 6(b)).

Quantitative analysis of neurofilament expression performed on low magnification fluorescence images (representative fields are provided in Supplemental Data, Figure S3) revealed that ~3% of the cells predifferentiated for 12 h and exposed to 1 g had NF-positive neurites. When cells were exposed to 50 g and 150 g, higher percentages were found (~20% and ~30% of the cells had NF-positive neurites, resp.; *P* < 0.05 in both cases).

In cells predifferentiated for 72 h and exposed to 1 g, ~10% of the cells had NF-positive neurites. When cells were exposed to 50 and 150 g, higher percentages were found (15% and ~40% of the cells had NF-positive neurites, resp.; *P* < 0.05 in both cases).

## 4. Discussion

Altered gravity can exert a deep influence on cellular behavior, on tissue functions, and even on whole organisms. Traditionally, microgravity has been investigated mainly because of its dramatic effects on human anatomy and physiology even after a short-term permanence in space [3]. On the other hand, it has been observed that microgravity is an effective means to obtain* in vitro* constructs that could be useful for a wide range of applications [23, 27]. The application of hypergravity to cell cultures for tissue engineering purposes remains instead relatively unexplored.

To the best of our knowledge, a review of the available literature evidences no significant data about effects on PC12 cells and, more generally, on neuronal cells after exposure to hypergravity. Only one paper reported on SH-SY5Y neuroblastoma cell line behavior after exposure to moderate hypergravity for short periods of time [33].

In the present work, the application of hypergravity conditions to PC12 neuron-like cells resulted in a modest but significant increase of metabolic activity.

In our study, altered gravity conditions were found to affect the transcriptional patterns of genes involved in neuronal maturation depending on the predifferentiation period. The upregulation of neurofilament-66 gene transcription upon hypergravity stimulation could be related to the finding of a different spatial expression pattern of the neurofilament-66 protein. The latter was mostly found at distal portions of the neurites rather than in the cell body, which is typical of a more advanced differentiation state [37, 38].

Above all, PC12 phenotype development resulted strongly affected by the hypergravity treatments, being neurites significantly longer after stimulation at high acceleration values.

Concerning effects of microgravity on neuronal cells, it has been reported that 1 h exposure induced damages in rodent neurons, resulting in a diminished neurite network density and neurite size, and in the alteration of *β*3-tubulin localization [36]. Alterations of the NGF and BDNF signaling pathways in cortical neurons were also found under simulated microgravity [39]. When PC12 cells were exposed to a middle-term duration (8 days) microgravity, a reduced neurite extension with respect to 1 g cultures was demonstrated as well [40]. Other evidences in the literature can be found concerning different cell lines that showed how microgravity affects cytoskeletal proteins and generally implies alterations of their normal structuring. For instance, an investigation on primary osteoblasts suggested how microtubules undergo a frustrated growth or precocious truncation in the presence of microgravity conditions [8]. Another cytoskeletal protein (actin) was found to be widely disorganized, with thickenings at the cell cortex upon exposure to microgravity in human umbilical vein endothelial cells [29].

Our findings concerning exposure of PC12 cells to different hypergravity values showed no qualitative alterations of cytoskeletal f-actin localization with respect to the controls. Our previous study on C2C12 skeletal myoblasts instead showed an increase in cytoplasmic actin filament thickness [31]. By contrast, the literature reports that microtubules (composed of *α*- and *β*-tubulin) are not affected by the exposure to 2 g hypergravity for 6 min in SH-SY5Y cells [30].

As previously mentioned, another interesting finding in our study is represented by the different localization of neurofilament-66 within the cells, depending on the stimulation. One of the most pronounced effect of hypergravity on PC12 cells indeed resulted to be neurofilament-66 localization in distal ends of the emitted neurites rather than in the cell bodies (as shown by control cultures). Being neurofilaments involved in the definition of the axonal shape [41, 42], this result seems to suggest that hypergravity may favor neurite development. Taken together, all these findings suggest that a short-term treatment with hypergravity is able to accelerate the differentiation process to an extent typical of longer differentiation times, and thus to promote a faster maturation of PC12 cells toward a neuronal phenotype.

Mechanisms involved in these phenomena are still to be clarified; however, we can hypothesize as hypergravity may act as a mechanical cue able to elicit neuronal development. It is in fact already widely recognized as a mechanical stress, such as stretching in SH-SY5Y cells [43] or shear stress in PC12 cells [44], which can induce an improvement in neurite sprouting. Increased gravity force could thus represent a further physical approach able to trigger signaling pathways (still to be elucidated), and thus to foster cellular responses typical of a mechanical stimulation.

In summary, the obtained results suggest that hypergravity can be considered as a physical cue able to improve neuron-like cell response in terms of differentiation. By driving cell behaviour, hypergravity could represent a simple, safe, and cost-effective approach in the biomedical field that could be used in the* in vitro* preparation of constructs for tissue engineering and drug screening [45–47] and that could also be considered for futuristic preclinical approaches in the treatment of pathological conditions [48].

## 5. Conclusion

Hypergravity was found to impact on PC12 neuron-like cell differentiation, accelerating neurite emission and increasing neurite length. These findings suggest that a treatment under high gravity conditions could represent a straightforward approach for those applications requiring a well-sustained neurite regeneration for a prompt recovery of function, like, for instance, tissue engineering of the peripheral nervous system. Of course, further studies are necessary to elucidate the role of hypergravity in addressing cell behavior, in particular by investigating its effects on the expression of differentiation markers at a protein level and on the activation of specific signaling pathways. Functional studies as well as the identification of suitable human cell sources are also mandatory for hypergravity treatments to be applied in human healthcare. Current results however provide encouraging evidences on the use of hypergravity as a physical stimulus suitable for the preparation of constructs for regenerative medicine purposes.

## Supplementary Material

Figure S1. reports additional fluorescence microscopy images (ß3-tubulin immunostaining) taken at low magnification to show PC12 cell neurite outgrowth and extension after hypergravity treatments.
Figure S2. depicts the distributions of neurite lengths measured on different samples exposed to hypergravity.
Figure S3. shows additional low magnification immunofluorescence microscopy images of PC12 cells presenting different localization of the differentiation marker neurofilament-66 after hypergravity treatments.

## Figures and Tables

**Table 1:** Primer sequences for qRT-PCR analysis.

Gene	Sequences
Glyceraldehyde 3-phosphate dehydrogenase (*Gapdh*)	F: 5′-AACCTGCCAAGTATGATGAC-3′ R: 5′-GGAGTTGCTGTTGAAGTCA-3′

Neurofilament-66 (*Ina*)	F: 5′-AGGCTGGAAGGTAAACTCAGAC-3′ R: 5′-CAATTCCAGGAGTGAAGCAGGA-3′

*β*3-tubulin (*Tubb*)	F: 5′-ATTCTGGTGGACCTGGAG-3′ R: 5′-CACTCTGACCGAAGATAAAGTT-3′
