# Supplementary material for: Hypergravity Stimulation Enhances PC12 Neuron-Like Cell Differentiation
Source: Biomed Res Int. 2015 Feb 16;2015:748121. doi: 10.1155/2015/748121 (PMC4345237; doi:10.1155/2015/748121)
Supplement: Supplementary file 1 — Figure S1. reports additional fluorescence microscopy images (ß3-tubulin immunostaining) taken at low magnification to show PC12 cell neurite outgrowth and extension after hypergravity treatments. Figure S2. depicts the distributions of neurite lengths measured on different samples exposed to hypergravity. Figure S3. shows additional low magnification immunofluorescence microscopy images of PC12 cells presenting different localization of the differentiation marker neurofilament-66 after hypergravity treatments. [file 748121.f1.pdf]

Hypergravity stimulation enhances PC12 neuron-like cell differentiation

Giada Graziana Genchi[1,*], Francesca Cialdai[2], Monica Monici[2], Barbara Mazzolai[1], Virgilio Mattoli[1] and Gianni Ciofani[1,*]

[1]Istituto Italiano di Tecnologia, Center for Micro-BioRobotics @SSSA, Viale Rinaldo Piaggio 34, 56025 Pontedera (Pisa), Italy

[2]ASAcampus Joint Laboratory, ASA Research Division, Department of Experimental and Clinical Biomedical Sciences, University of Florence, Viale Pieraccini 6, 50139 Florence, Italy

[*]giada.genchi@iit.it, gianni.ciofani@iit.it

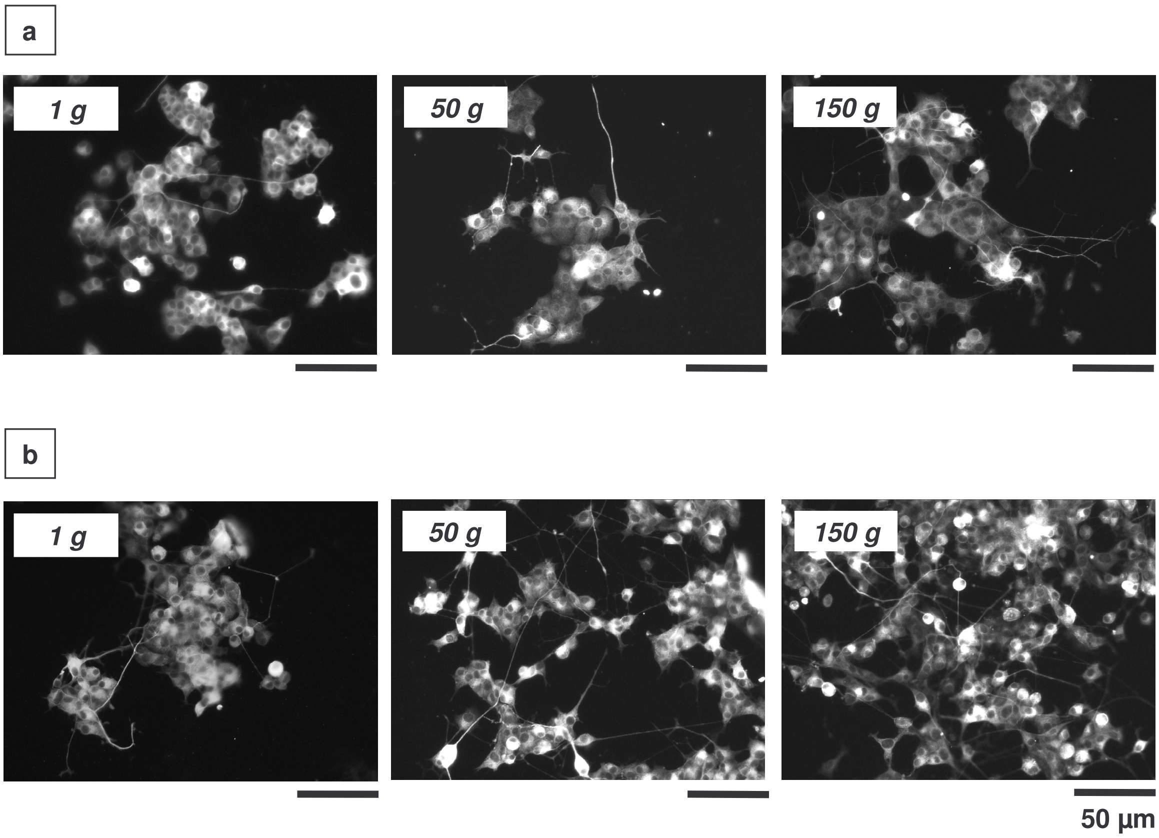

Figure S1. Immunofluorescence staining of β3-tubulin in PC12 cell cultures pre-differentiated for 12 h (**a**) and 72 h (**b**). The staining was performed after 48 h from hypergravity treatment.

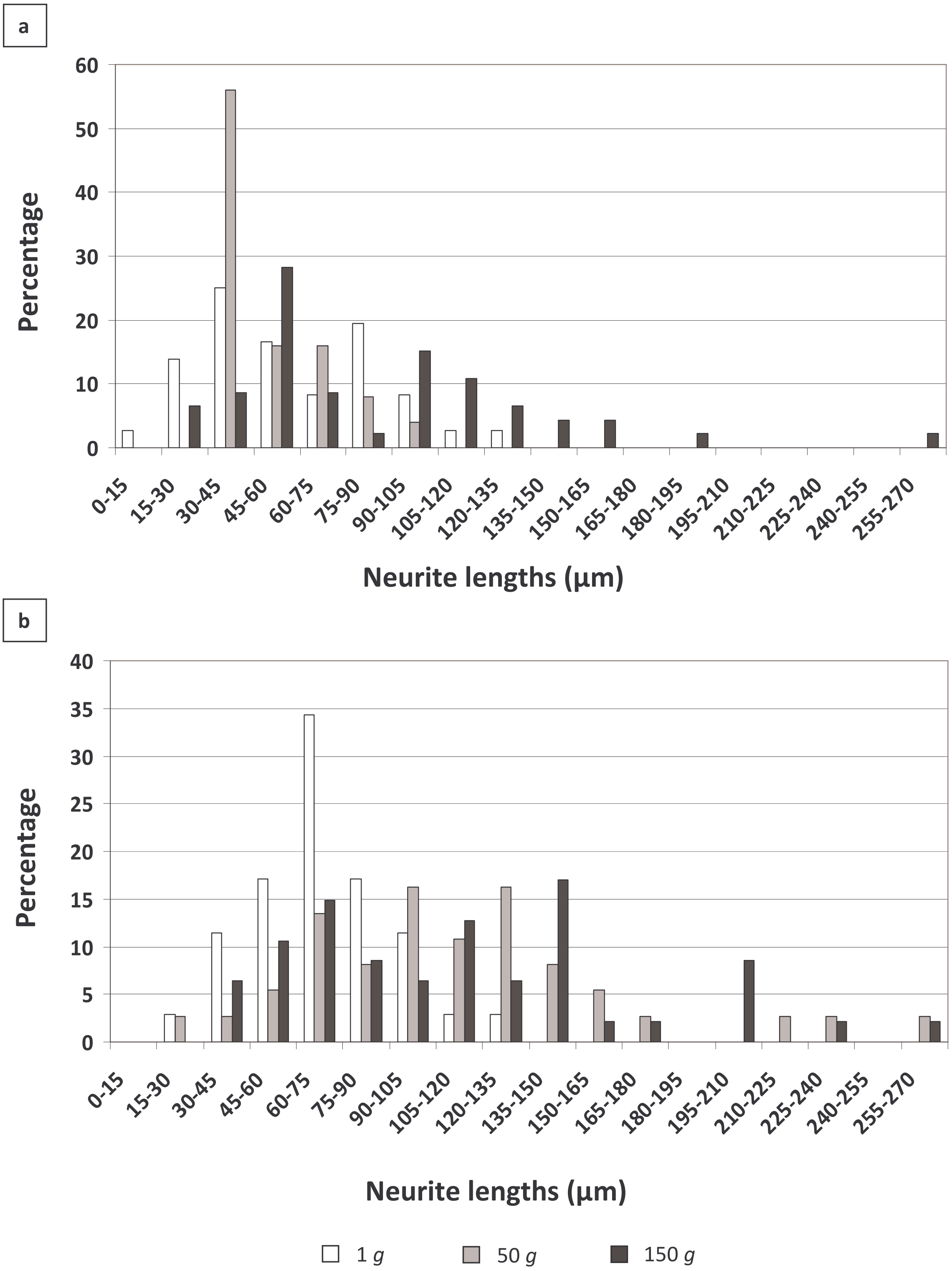

Figure S2. Neurite length distributions in PC12 cell cultures pre-differentiated for 12 h (**a**) and 72 h (**b**). The lengths were measured after 48 h from hypergravity treatment.

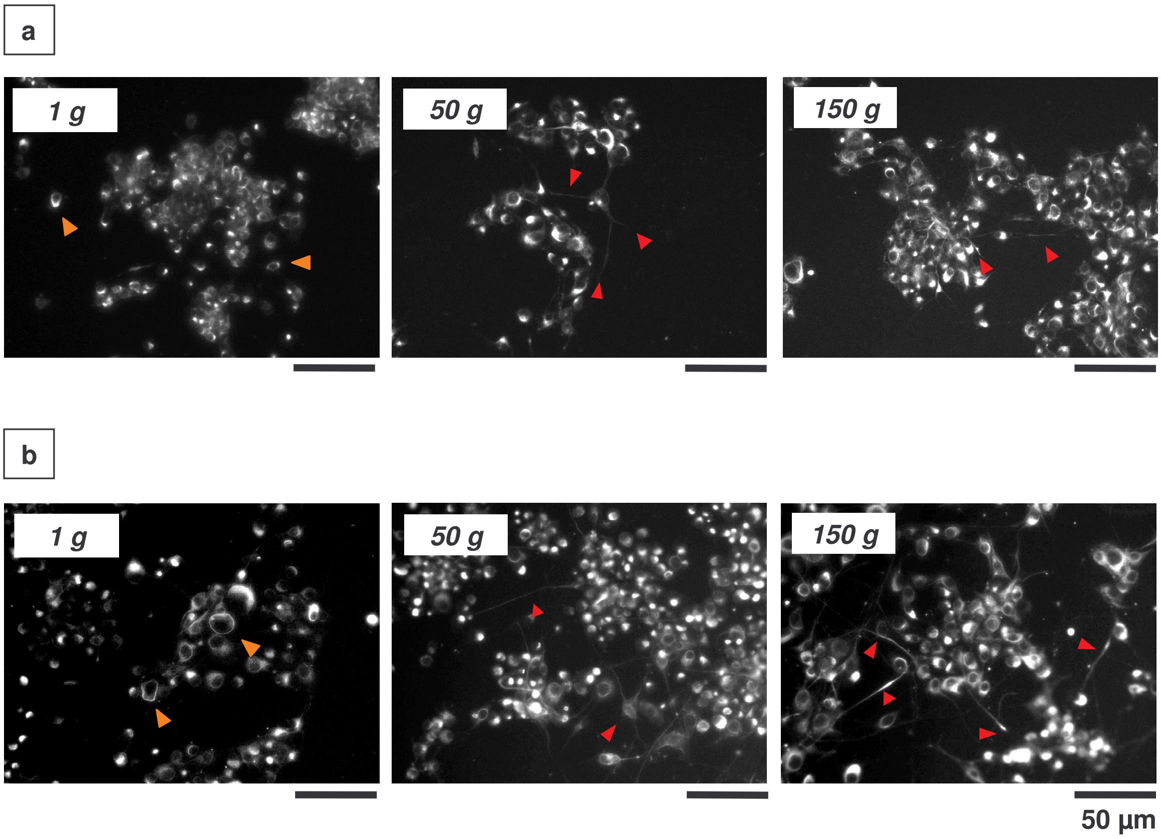

Figure S3. Immunofluorescent staining of neurofilament-66 in PC12 cell cultures pre-differentiated for 12 h (**a**) and 72 h (**b**). The staining was performed after 48 h from hypergravity treatment. Arrows evidence neurofilament-66 organization in a ring-like structure in control cultures, whereas the marker is localized in neurites and in growth cones of hypergravity-stimulated cells.
